# Association between single nucleotide polymorphism rs11057401 of CCDC92 gene and the risk of coronary heart disease (CHD)

**DOI:** 10.1186/s12944-018-0672-1

**Published:** 2018-02-13

**Authors:** Lingyan Xiao, Dongyang Shi, Hui Zhang, Yanchun Zhang, Yongfu Liu, Hu Lu, Yishan Zheng

**Affiliations:** 1 Intensive care unit, the Second Hospital of Nanjing, Southeast University, Nanjing, Jiangsu 210008 China; 2 Department of Endocrinology, the Affiliated Hospital of Xuzhou Medical University, Xuzhou, Jiangsu 221003 China; 3 Department of Cardiology, the Second Hospital of Huai’an, Xuzhou Medical University, Huai’an, Jiangsu 223002 China

**Keywords:** CCDC92 gene, Single nucleotide polymorphism, Lipids, Coronary artery disease, Ischemic stroke

## Abstract

**Background:**

Given that the CCDC92 (coiled-coil domain containing 92) was important in insulin resistance, we sought to investigate whether the CCDC92 rs825476 SNP is associated with the risk of CHD in Chinese Han population.

**Methods:**

Rs11057401 was genotyped for 817 patients with CHD and 724 age- and sex-matched controls using PCR-based Invader assay with the probe sets designed and synthesized by third wave.

**Results:**

Patients were found to have a significantly higher frequency of AA than the controls (23.5% vs. 14.7%, OR = 1.60, *p* = 0.000), and the frequency of allele A was found to be remarkably higher in the patients than the controls (48.1% vs. 40.3%, OR = 1.19, *p* = 0.000). Multivariate logistic analysis showed that the incidence of CHD was positively correlated with hyperlipidemia, T2D and rs11057401 AA/AT genotypes. The FPG, TC, and ApoA1 levels in the CHD patients were different among the AA, AT and TT genotypes (*P* < 0.05), the A allele carriers had higher FPG, TC and lower ApoA1 levels than the A allele non-carriers (*P* < 0.05).

**Conclusion:**

The genotypic and allelic frequencies of the rs11057401 SNP were significantly different between the patients with CHD and controls. Subjects with AA genotype or A allele were associated with an increased risk of CHD. The AA/AT genotypes were also associated with increased serum FPG, TC and decreased ApoA1 in CHD.

## Background

Coronary artery disease (CAD) is one of the most common cardiovascular disease, with high morbidity and mortality [1]. Coronary heart disease (CHD) is the most severe clinical manifestation of CAD and the leading cause of death worldwide [2]. CHD is a complex disease characterized by the inheritance of multiple genetic variants in addition to environmental factors which worsen the disease state [3, 4]. Many factors have been associated with CHD, including plasma lipid concentrations, blood pressure, smoking, diabetes and markers of inflammation. There has long been evidence from observational studies that those with type 2 diabetes (T2D) have an increased risk of CHD [5]. Patients with T2D are at a twofold higher risk of mortality due to CHD as compared to individuals who do not have T2D [6]. Recent Genome-wide association studies have also identified several loci associated with both T2D and CHD risk [7]. Insulin resistance plays an important role in the pathogenesis and evolution of T2D [8]. Similarly, insulin resistance has also been found to be the single most important cause of CHD, responsible for about 42% of myocardial infarction [9, 10]. Coiled-coil domain containing 92 (CCDC92) is a coiled-coil domain protein that interacts with proteins at the centriole–ciliary interface, and is found to be a previously unrecognized molecule influencing adipocyte differentiation [7]. Given that the CCDC92 was important in insulin resistance, we sought to investigate whether CCDC92 plays role in the development of CHD. The aim of this study was to determine whether the CCDC92 rs825476 SNP is associated with the risk of CHD in Chinese Han population.

## Methods

### Subjects

**Fig. 1:**
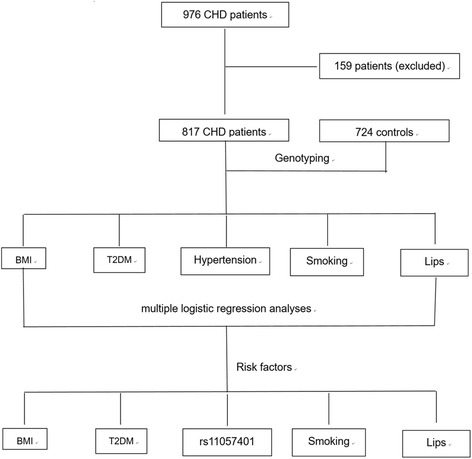
Flow chart of this study

A total of 976 patients with CHD and 724 age- and sex-matched controls were recruited. CHD can be defined as including typical ischemic symptoms, plus one or more electrocardiographic changes (ST-segment depression or elevation of ≥0.5 mm, T-wave inversion of ≥3 mm in ≥3 leads, or left bundle branch block), in addition to increases in cardiac markers, such as creatinine kinase-MB and troponin T. Coronary angiography was carried out in patients with CHD. The CHD subjects who had significant coronary stenosis (≥ 50%) in at least one of the three main coronary arteries or their major branches (branch diameter ≥ 2 mm) were included in the study. In addition, the angiographic severity of disease was classified according to the number of coronary vessels with significant stenosis (luminal narrowing ≥50%) as one-, two-, or three-vessel disease in the three major coronary arteries. All participants were unrelated Chinese and underwent biochemical testing. Patients were excluded when they had autoimmune disease, or serious chronic diseases or a history of CHD. Finally, a total of 817 patients with CHD were included (Fig. 1). In 817 patients, 243 were Non-ST elevation myocardial infarction (NSTEMI), 122 ST elevation myocardial infarction (NSTEMI), 201 unstable angina and 251 stable angina pectoris. The study was in accordance with the principles of the Declaration of Helsinki and was approved by the clinical trials and biomedical ethics committee of the Second Hospital of Nanjing, Southeast University. Written informed consent for participation in the study and the donation of samples were obtained from all participants and their legal proxies.

### Biochemical analysis

Venous blood samples were collected from all subjects after at least 12 h of fasting. The following information including fasting plasma glucose (FPG, mmol/L), triglycerides (TG, mmol/L), total cholesterol (TC, mmol/L), low-density lipoprotein cholesterol (LDL-C, mmol/L), high-density lipoprotein cholesterol (HDL-C, mmol/L), Apolipoprotein A1 (ApoA1, g/L), Apolipoprotein B (ApoB, g/L) smoking habits, and chronic diseases containing hypertension and T2D. FPG and lipid/lipoprotein levels were determined using standard laboratory techniques. The diagnosis of hypertension was made if patients were under treatment or the mean blood pressure of 3 measurements was > 140/90 mmHg. T2DM was diagnosed according to the World Health Organization criteria or had a documented clinical diagnosis of T2DM from medical records. The individuals with TC > 5.17 mmol/L and/or TG > 1.70 mmol/L were defined as hyperlipidemic.

### Genotyping of SNP

Rs11057401 was genotyped using PCR-based Invader assay with the probe sets designed and synthesized by third wave. The genotyping results were read with an ABI PRISM7900HT sequence detection system (Applied Biosystems, Foster City, CA). Twenty percent of the samples were selected randomly to validate the reproducibility of the genotyping results.

### Statistical analysis

Data were analyzed using SPSS software (SPSS Inc., Chicago, IL, USA, version 13.0). Chi square test was used to examine differences of allele frequencies and genotype distributions between CHD patients and controls. Unconditional multiple logistic regression analyses were employed to estimate unadjusted and adjusted odds ratios (ORs) and 95% confidence intervals (CIs). Adjusted ORs and *P* values were corrected for factors associated with CHD that included sex, age, body mass index (BMI) and smoking status, T2D, hypertension and hyperlipidemia. The association between genotypes and clinical parameters was tested by analysis of covariance (ANCOVA). Statistical significance was determined by a *P* value < 0.05. All *P* values were two-sided.

## Results

### General characteristics

The levels of FPG, TC, LDL-C, and the percentages of T2D and hypertension were higher but the concentrations HDL-C, ApoA1 and the ApoA1 to ApoB ratio were lower in persons who suffered from CHD than in controls (*P* < 0.05). There were no significant differences of TG, ApoB, BMI, and smoking habits between the two groups (*P* > 0.05) (Table 1).

**Table 1:** Comparison of general characteristics between controls and CHD patients

	CHD	Control	*P*
Male/Female	571/246	523/201	0.311
Age (years)	64.3 ± 10.6	63.7 ± 11.2	0.428
BMI (kg/m^2^)	23.7 ± 3.1	22.9 ± 3.2	0.572
FPG (mmol/L)	6.42 ± 1.57	6.15 ± 1.43	0.000
TC (mmol/L)	4.57 ± 1.21	4.49 ± 0.62	0.001
TG (mmol/L)	1.53 ± 1.64	1.62 ± 1.37	0.327
LDL-C (mmol/L)	2.97 ± 0.68	2.74 ± 0.82	0.000
HDL-C (mmol/L)	1.20 ± 0.43	1.89 ± 0.39	0.000
ApoA1 (g/L)	1.03 ± 0.27	1.42 ± 0.35	0.000
ApoB (g/L)	1.01 ± 0.25	0.97 ± 0.36	0.312
ApoA1/ApoB	1.01 ± 0.44	1.57 ± 0.57	0.027
Smoking, n (%)	296 (36.2%)	237 (33.6%)	0.273
T2DM, n (%)	180 (22.0%)	123 (17.0%)	0.013
Hypertension, n (%)	355 (43.5%)	240 (33.1%)	0.000

*LDL-C* Low-density lipoprotein cholesterol, *HDL-C* High-density lipoprotein cholesterol, *TC* Total cholesterol, *TG* Triglyceride, *FPG* fasting plasma glucose, *ApoA1* Apolipoprotein A1, *ApoB* Apolipoprotein B

### Genotypic and allelic frequencies

No significant difference regarding the genotype frequency in the patients and the controls was showed by HWE test. Patients were found to have a significantly higher frequency of AA than the controls (23.5% vs. 14.7%, OR = 1.60, *p* = 0.000), and the frequency of allele A was found to be remarkably higher in the patients than the controls (48.1% vs. 40.3%, OR = 1.19, *p* = 0.000). (Table 2).

**Table 2:** Allele and genotype frequencies of rs11057401 for CHD patients and normal controls

Genotype	CHD		Control		χ2(*P*)	Allele	CHD		Control		χ2(*P*)
	N	%	N	%			n	%	n	%	
AA	175	23.5	103	14.7	0.000	A	680	48.1	566	40.3	0.000
AT	330	44.3	360	51.2		T	734	51.9	840	59.7	
TT	202	32.2	240	34.1							

### Related risk factors for CHD

Multivariate logistic analysis showed that the incidence of CHD was positively correlated with hyperlipidemia, T2D and rs11057401 AA/AT genotypes (Table 3).

**Table 3:** The relative risk factors for CHD

Relative factors	OR (95% CI))	*P*
Male	1	
Female	1.10 (0.95–1.27)	0.418
Age < 60 years	1	
Age ≥ 60 years	1.14 (0.93–1.31)	0.362
Nonsmoking	1	
Smoking	1.73 (1.26–2.34)	0.009
BMI < 24 kg/m^2^	1	
BMI ≥ 24 kg/m^2^	1.95 (1.46–2.50)	0.006
rs11057401 TT	1	
rs11057401 AA/AT	1.61 (1.21–2.16)	0.012
No T2DM	1	
T2DM	1.52 (1.17–1.93)	0.017
Normotensive	1	
Hypertension	1.17 (0.92–1.47)	0.383
Normal blood lips	1	
Hyperlipidemia	2.13 (1.73–2.81)	0.005

*LDL-C* Low-density lipoprotein cholesterol, *HDL-C* High-density lipoprotein cholesterol, *TC* Total cholesterol, *TG* Triglyceride, *FPG* fasting plasma glucose, *ApoA1* Apolipoprotein A1, *ApoB* Apolipoprotein B

### Genotypes and clinical parameters

The FPG, TC, and ApoA1 levels in the CHD patients were different among the AA, AT and TT genotypes (*P* < 0.05), the A allele carriers had higher FPG, TC and lower ApoA1 levels than the A allele non-carriers (*P* < 0.05) (Table 4).

**Table 4:** Clinical characteristics of the patients according to the rs11057401 polymorphism in the CCDC92 gene

	FPG (mmol/L)	TC (mmol/L)	TG (mmol/L)	LDL-C (mmol/L)	HDL-C (mmol/L)	ApoA1 (g/L)	ApoB (g/L)	ApoA1 /ApoB
TT	6.29 ± 1.62	4.40 ± 1.16	1.51 ± 1.72	2.95 ± 0.70	1.21 ± 0.46	1.05 ± 0.31	1.02 ± 0.28	1.03 ± 0.49
AT	6.35 ± 1.47	4.56 ± 1.37	1.54 ± 1.62	2.98 ± 0.65	1.22 ± 0.41	1.04 ± 0.25	1.01 ± 0.21	1.02 ± 0.42
AA	6.47 ± 1.51	4.63 ± 1.04	1.55 ± 1.54	2.99 ± 0.66	1.19 ± 0.45	1.01 ± 0.26	0.98 ± 0.24	0.99 ± 0.43
P	0.021	0.026	0.371	0.392	0.427	0.038	0.621	0.205
TT	6.29 ± 1.62	4.40 ± 1.16	1.51 ± 1.72	2.95 ± 0.70	1.21 ± 0.46	1.05 ± 0.31	1.02 ± 0.28	1.03 ± 0.49
AA+AT	6.42 ± 1.48	4.61 ± 1.27	1.54 ± 1.59	2.98 ± 0.67	1.20 ± 0.39	1.02 ± 0.24	1.00 ± 0.25	1.00 ± 0.43
P	0.038	0.017	0.231	0.428	0.518	0.029	0.462	0.379

*LDL-C* Low-density lipoprotein cholesterol, *HDL-C* High-density lipoprotein cholesterol, *TC* Total cholesterol, *TG* Triglyceride, *FPG* fasting plasma glucose, *ApoA1* Apolipoprotein A1, *ApoB* Apolipoprotein B

## Discussion

Reduced insulin-stimulated glucose metabolism in skeletal muscles (insulin resistance) and hyperinsulinism have been found the common features in a widespread of diseases, being implicated in adverse health outcomes such as hypertension, cardiovascular disease, cerebrovascular disease, peripheral vascular disease (atherosclerosis), congestive heart failure, non-alcoholic fatty liver disease, android obesity and a variety of malignancies [11, 12]. After correction for traditional risk factors like hypertension, hyperlipidaemia and family history, insulin resistance has been considered as an independent risk factor for ischemic heart diseases [13, 14] and represent a major underlying abnormality driving cardiovascular diseases [15].

The pathogenic mechanism by which insulin resistance is thought to induce CHD is said to be through its direct atherogenic action on vessel wall cells and indirectly through upper body obesity and lipids homeostasis [16]; and it has been associated with higher levels of triglycerides, lower levels of HDL-C, elevated blood pressure, non-insulin dependent diabetes mellitus (NIDDM) [17]. A recent GWAS study showed that genetically mediated increase in T2D risk also confers higher CHD risk [7]. Moreover, joint T2D–CHD analysis identified eight variants—two of which are coding—where T2D and CHD associations appear to colocalize, including a new joint T2D–CHD association at the CCDC92 locus that also replicated for T2D. Klarin et al. [8] performed a GWAS in UK Biobank testing ~ 9 million DNA sequence variants for association with coronary artery disease and carried out meta-analysis with previously published results, identifying 15 new loci. They observed significant association for CCDC92 p.Ser70Cys (rs11057401) with body fat percentage and waist-to-hip circumference ratio, as well as plasma HDL, triglyceride, and adiponectin levels. The directionalities of these associations are hallmarks of insulin resistance and lipodystrophy, and the association with plasma adiponectin levels localizes these genetic effects to adipose tissue. Chasman et al. [18] conducted a GWAS, and demonstrated genetic influences of CCDC92 gene on concentration and size of LDL, HDL, and VLDL particles. Lotta et al. [19] conducted an integrative genomic analysis providing evidence that loci of CCDC92 gene influence adipose gene expression, leading to impaired adipogenesis, reduced peripheral fat depots and, ultimately, increased risk of cardiac disease. The aforementioned studies have strongly indicated the important role of CCDC92 in insulin resistance, which would further influence CHD risk. In addition to genetic factors, nutraceuticals play a bigger role on dyslipidemia and cardiovascular diseases. Nutraceuticals and functional food ingredients that are beneficial to vascular health may represent useful compounds that are able to reduce the overall cardiovascular risk induced by dyslipidemia by acting parallel to statins or as adjuvants in case of failure or in situations where statins cannot be used [20].

Our study demonstrated that the CCDC92 gene could influence the levels of serum lipid and glucose, which would add the risks of CHD. Patients with A allele had higher levels of FPG, TC, and lower ApoA1 as well as increased risks of CHD when compared to those with T allele. In addition to abnormal lipid metabolism, insulin resistance was also an important risk factor for CHD. Previous studies have repeatedly approved the role of abnormal lipid metabolism in the development of CHD. Our study revealed that insulin resistance related gene also pose effects on CHD, implying shared etiological pathways of CHD with T2D.

## Conclusions

This study revealed that the genotypic and allelic frequencies of the rs11057401 SNP were significantly different between the patients with CHD and controls. Subjects with AA genotype or A allele were associated with an increased risk of CHD. The AA/AT genotypes were also associated with increased serum FPG, TC and decreased ApoA1 in CHD.

## Abbreviations

ANCOVA: Analysis of covariance
Apo: Apolipoprotein
ApoA1: Apolipoprotein A1
ApoB: Apolipoprotein B
BMI: Body mass index
CCDC92: Coiled-coil domain containing 92
CHD: Coronary heart disease
FPG: Fasting plasma glucose
GWAS: Genome-wide association study
HDL-C: High-density lipoprotein cholesterol
HWE: Hardy-Weinberg equilibrium
LDL-C: Low-density lipoprotein cholesterol
PCR: Polymerase chain reaction
SNP: Single nucleotide polymorphism
T2DM: Type 2 diabetes mellitus
TC: Total cholesterol
TG: Triglyceride
